# Perspectives on the role of stakeholders in knowledge translation in health policy development in Uganda

**DOI:** 10.1186/1472-6963-13-324

**Published:** 2013-08-19

**Authors:** Juliet Nabyonga Orem, Bruno Marchal, DavidKaawa Mafigiri, Freddie Ssengooba, Jean Macq, Valeria Campos Da Silveira, Bart Criel

**Affiliations:** 1 WHO Uganda office, Health systems and services cluster, P. O. Box 24578, Kampala, Uganda; 2 Institute of Tropical Medicine Antwerp-Belgium, Nationalestraat 155, Antwerp, 2000, Belgium; 3 Makerere University School of Social Sciences, P.O Box 7072, Kampala, Uganda; 4 Makerere University, School of Public Health, Kampala, P.O. Box. 7072, Uganda; 5 Université Catholique de Louvain, Boite 3058, Clos Chapelle aux champs, 30, Bruxelles, 1200, Belgium

**Keywords:** Research, Uptake, Policy, Practice, Roles, Stakeholders, Uganda, Low-income countries

## Abstract

**Background:**

Stakeholder roles in the application of evidence are influenced by context, the nature of the evidence, the policy development process, and stakeholder interactions. Past research has highlighted the role of stakeholders in knowledge translation (KT) without paying adequate attention to the peculiarities of low-income countries. Here we identify the roles, relations, and interactions among the key stakeholders involved in KT in Uganda and the challenges that they face.

**Methods:**

This study employed qualitative approaches to examine the roles of and links among various stakeholders in KT. In-depth interviews were conducted with 21 key informants and focused on the key actors in KT, their perceived roles, and challenges.

**Results:**

Major stakeholders included civil society organizations with perceived roles of advocacy, community mobilization, and implementation. These stakeholders may ignore unconvincing evidence. The community’s role was perceived as advocacy and participation in setting research priorities. The key role of the media was perceived as knowledge dissemination, but respondents noted that the media may misrepresent evidence if it is received in a poorly packaged form. The perceived roles of policy makers were evidence uptake, establishing platforms for KT and stewardship; negative roles included ignoring or even misrepresenting evidence that is not in their favor. The roles of parliamentarians were perceived as advocacy and community mobilization, but they were noted to pursue objectives that may not be supported by the evidence. The researchers’ main role was defined as evidence generation, but focusing disproportionately on academic interests was cited as a concern. The donors’ main role was defined as funding research and KT, but respondents were concerned about the local relevance of donor-supported research. Respondents reported that links among stakeholders were weak due to the absence of institutionalized, inclusive platforms. Challenges facing the stakeholders in the process of KT were identified.

**Conclusions:**

Our investigation revealed the need to consider the roles that various stakeholders are best placed to play. Links and necessary platforms must be put in place to achieve synergy in KT. Relevant capacities need to be built to overcome the challenges faced by the various stakeholders.

## Background

Although efforts have been made to improve uptake of evidence in the development of public health policy, much remains to be done, especially in low-income countries (LICs) [1-3]. Several terms have been used for the notion of uptake of evidence in health policy development, including knowledge translation (KT), research utilization, evidence-based decision making, and getting research into policy and practice. In this article, we use KT as an all-encompassing term defined as “*a dynamic and iterative process that includes synthesis, dissemination, exchange, and ethically sound application of knowledge to improve health, provide more effective health services and products, and strengthen the health care system*” [4].

There is an extensive body of literature on the factors that facilitate or hinder the uptake of evidence and; several models for KT have been proposed [5-9]. Sauerborn *et al.* highlighted the role of stakeholders in KT, stating that evidence can only be relevant if it is known and used by stakeholders [10]. The current study builds upon an earlier study on KT in Uganda in which we developed a middle range theory (MRT) of how the uptake of evidence in health policy development can be improved [11]. MRTs should be understood here as defined by Merton in 1968 as “*theories that lie between the minor but necessary working hypotheses (…) and the all-inclusive systematic efforts to develop a unified theory that will explain all the observed uniformities of social behavior, social organization, and social change*” [12]. The themes in our MRT that were previously identified by policy actors as most important were: (1) institutional strengthening for KT to enhance ownership and better application of evidence; (2) timely provision of high-quality, relevant evidence produced by credible researchers; and (3) partnerships involving all relevant stakeholders, including communities, throughout the process of knowledge generation up to its application [11]. In this article, we focus on the partnerships, roles, relations, and interactions of key stakeholders involved in KT. We define partnership as a complementary relationship that enhances attainment of a given objective and in which the roles of the various stakeholders are well identified [6].

Potential stakeholders in KT encompass both the public and private sectors and have been identified as government policy makers, politicians, service providers, health managers, professional bodies and networks, knowledge brokers, donors, communities, civil society organizations (CSOs)/non-governmental organizations, and the media. The literature highlights a multiplicity of potential roles that can be played by the various stakeholders in KT. For example, CSOs have the capacity to mobilize communities to demand policy change, to contribute to all stages of research, to mobilize resources, and to disseminate and utilize evidence in implementing their own programs [13-15]. CSOs are here defined as organized groups concerned with public interests, including non-governmental organizations and less formally organized groups based in local communities [14]. However, the challenges faced by CSOs include inadequate capacity to navigate the political terrain and to influence policy, the service-delivery focus of their operations, financial constraints, limited capacity to undertake research beyond evaluation of their programs and pilot programs, weak links with the research community and dependence on donors [7,16,17]. Communities have also been identified as stakeholders because their involvement facilitates the generation of research results that are more responsive to local needs [6,18]. However, communities are challenged by a lack of relevant institutional structures, the weak capacity of communities, and the costs associated with enabling their participation [19]. Although Armstrong *et al.* maintain that the roles of CSOs, the media, and pressure groups in KT remain poorly understood and unexplored [20], the media have been shown to enjoy major influencing power; their roles in KT have been stated to be dissemination of information and social mobilization [10,21,22].

Policy makers influence the degree to which research informs policy development, shape the research prioritization process, and impact the actual generation of knowledge [7,21]. In addition, policy makers play a key role in establishing the required platforms for engagement in KT and in building partnerships between researchers and other stakeholders [23]. The roles of politicians have been identified as mobilization of communities, dissemination of evidence, and advocacy. However, politicians may face several challenges, including the pressure to respond to their constituencies and political ideological agendas that may influence how they deal with the available evidence [3,10].

Although researchers intervene as major stakeholders in the process of KT [10,21,24], they have been criticized for failing to address policy-relevant questions, for lacking knowledge of the policy process, for poor packaging of results, and for possessing limited dissemination skills [18,21,25,26].

The donors’ main role is funding research, KT activities, and implementation of research findings [3,21]. However, Young pointed out that the role of donors in KT can be both supportive and disruptive [27]. Failure to address local research priorities and taking control of the research and policy agendas are among the criticisms leveled against donors [28,29].

Knowledge brokers are defined as people who link the various stakeholders through their capacity to disseminate research in user-friendly packages. However, researchers have raised concerns regarding the need to evaluate their effectiveness [7,30,31]. Formal networks can bring together people with a common interest into tightly organized platforms; informal networks of stakeholders are also common. If information flows freely within these networks, it is more easily taken up in policy development [28,32,33], but some researchers have pointed out the complexity of the interactions in such networks [5,20]. Table 1 summarizes the roles played by various stakeholders according to the literature.

**Table 1:** A summary of the roles played by various stakeholders in KT

**Stakeholder**	**Roles**
CSOs	Representing and advocating for the communities they serve, mobilizing resources for undertaking research, undertaking research, disseminating and facilitating the implementation of decisions based on evidence
Communities	Involvement in setting the research agenda, demanding the application of evidence
Media	Dissemination of information and social mobilization
Policy makers	Identifying knowledge gaps, commissioning and guiding research processes, applying evidence in decision-making, establishing institutional platforms for KT
Politicians	Advocacy, setting research priorities, disseminating evidence, mobilizing communities
Researchers	Generating evidence
Donors	Providing funding for research, KT activities, and implementation of research findings
Knowledge brokers	Disseminating evidence
Formal and informal networks and professional bodies	Generating and disseminating evidence

Tomlinson *et al.* stated that the exact composition and configuration of the stakeholder groups is country-specific [34], and stakeholder roles in KT are influenced by the context in which the evidence, the policy development process, and the stakeholders interact. Past research has highlighted the role of stakeholders and the importance of sustained partnerships, but did not pay adequate attention to the peculiarities of LICs [10]. This investigation aims to fill this gap by developing a better understanding of the perceived roles of the key stakeholders in KT in Uganda.

We hypothesized that stakeholder interactions and relations influence the uptake of evidence in public health policy development. Effective partnerships often do not emerge spontaneously; links need to be established, a common vision built, capacities strengthened, and challenges/barriers addressed. By using a qualitative approach and involving key informants, we addressed the question of partnerships in KT and explored the perceived roles of the various stakeholders in KT in Uganda. We also evaluated whether and how these stakeholders engage in partnerships.

## Methods

This qualitative study utilized document review and in-depth interviews conducted between November 2010 and January 2011 to examine the roles and links among various stakeholders in KT, as related to public health policy^[a]^. A sampling frame of participants was developed by the research team, in part based on the initial findings of our document review, which identified various types of stakeholders. Study participants were then purposively selected who were deemed to possess relevant characteristics, experience, and/or knowledge about the research question [36]. Candidate respondents were chosen on the basis of their institutional affiliations, their seniority in their current roles, and/or their responsibilities as health policymakers. Overall, 15 members of the Health Policy Advisory Committee (HPAC) were interviewed. HPAC is the policy advisory organ of the health sector; its membership and number of representatives from each stakeholder group have been agreed on by all stakeholders in the health sector. Selected HPAC members included government officials at the central level (n = 4), service providers at the district level (n = 1), and representatives of CSOs including coordinators (n = 2) and service providers (n = 2). Additionally, representatives from private for-profit (n = 1) organizations, multilateral donors (n = 3), bilateral donors (n = 2), researchers (n = 2), journalists/media (n = 2), and parliamentarians (n = 2) were interviewed. Professional bodies, policy networks, and knowledge brokers were not selected because an earlier study on KT in Uganda indicated that these potential stakeholders did not have a significant role in KT in Uganda [11].

Officials in the HPAC are the most senior officers in their institutions/agencies. In the case of private not-for-profit and private for-profit organizations, HPAC members are delegated representatives of faith-based medical bureaus and private health providers, respectively. Districts and service providers are represented by officers from local government health services. Researchers were selected on the basis of their previous work on KT and their focus on health system development. Journalists were selected based on their focus on reporting on health issues; they were the most senior reporters on health issues. The politicians were members of the social service committee, which deals with health issues. Detailed information regarding the selected key informant stakeholders appears in Table 2.

**Table 2:** Key informant respondents

	**Stakeholder group**	**HPAC (n)**	**Selected (n)**	**Duration in post**
**Policy makers (n = 15)**	**Central level MoH**	9	4	three for more than 7 years and one for 3 years
	**Service providers and health managers at the service-provision level**			
	District level	1	1	for over 7 years
	Facility-based CSOs	2	2	at least 6 years
	Non-facility-based CSOs	2	2	6 years and 3 years
	Private for-profit providers	1	1	2 years
	**Donors**			
	Bilateral	4	2	Four for 6 years, one for 6 months, and one for 2 years
	Multilateral	3	3	For at least 7 years
	**Researchers**			
	Public (from the School of Public Health)	0	1	over 14 years
	Private (from a private research group)	0	1	2 years
	**Journalists (specialized health reporters)**	0	2	at least 10 years
	**Parliamentarians/politicians**	0	2	2 years, but previously worked with the health sector for over 15 years
	**Total**		**21**	

Researchers, journalists and parliamentarians are not members of HPAC.

JNO developed the interview guide, which consisted of open-ended questions intended to examine perceptions about the roles of various stakeholders in KT, the challenges faced by these stakeholders, and the availability of platforms for stakeholder engagement. The research team reviewed and refined the guide prior to pretesting it with volunteer colleagues in the Uganda office of the World Health Organization (WHO; n = 2), technical officers in the Ministry of Health (MoH; n = 2), and one researcher from the School of Public Health. Key informants were contacted and invited by email or telephone to participate in the study. All identified respondents agreed to participate and were interviewed.

All interviews were conducted by JNO, in English and face-to-face. Interviews were recorded, transcribed verbatim, and entered into Microsoft Word for editing as the first step in “formal” analysis. During the interviews, JNO made additional notes to record the initial findings and impressions that were used to augment the transcribed interviews. The interviews lasted 45 minutes on average. Deductive content analysis techniques were used to identify emerging categories linked to the research issues [37]. As a first step to analysis, JNO and DKM read all transcribed interviews and developed categories of emerging issues. The study team collaboratively analyzed the transcripts to identify categories according to the type of key informant, the emerging issues organized by the research areas defined in the interview guide, and by the various stakeholders in KT. Deductive content analysis was undertaken by JNO, BM, and DKM to assess how respondents perceived the role of the various stakeholders in KT and the challenges they face. Patterns in responses were compared with results from the literature regarding the roles for and challenges facing the various stakeholders to identify convergent and other emerging issues. JNO, BM, and DKM initially identified categories and emerging issues independently, after which JNO and DKM reviewed and interpreted the findings. Relying on manifest analytical approach, converging issues were again reviewed by the rest of the research team and where interpretation differed, consensus was achieved through revisiting the raw data and discussions. Where necessary, quotations that best represented emerging issues were edited slightly for flow, but the meaning of the text was preserved.

Informed consent was obtained from all respondents prior to the interviews. Study participants were informed about the purpose of the study and the scope of the issues in the in-depth interview guide. Confidentiality was ensured in data management, and only aggregate information without subject identifiers is reported. All data were secured in a safe location accessible only to the study team. Ethical approval was obtained from the Institutional Review Board of the Institute of Tropical Medicine, Antwerp, Belgium (IRB/AC/ac/197) and the Uganda National Council for Science and Technology (SS 2920).

## Results

The respondents were asked to identify the stakeholders in KT in Uganda and to report what they felt were the positive and negative roles played by these stakeholders (Table 3). Below we present specific issues that were identified for each group of stakeholders.

**Table 3:** Identified positive and negative roles for various stakeholders

	**Positive roles (no. of respondents)**	**Negative roles (no. of respondents)**	**Challenges faced in playing their role in KT**
CSOs	Using research results (10)	May de-campaign evidence if they are not convinced (2)	Lack of capacity, weak internal organization, lack of independence
	Advocating with policy makers to implement evidence (8)		
		If not given proper information, may cause confusion (1)	
	Mobilizing communities (7)		
	Disseminating research (2)		
	Undertaking research (2)		
	Liaising with the media (1)		
	Generating research topics (1)		
Communities	Can demand that evidence be implemented or demand that a policy be developed (6)	Can be disruptive if results do consider community-contextual issues (1)	Currently not able to engage in research policy processes
	Contributing to development of the research agenda (6)		
	Can participate in research (1)		
Media	Disseminating research results (15)	Misrepresenting evidence (5)	Not well organized and are communicating to a public that is not strong enough to respond
	Putting forward community views (4)	De-campaigning implementation of evidence if they are not convinced (3)	
	Advocating for implementing evidence (1)		
Policy makers	Using evidence in developing policies and implementation (12)	If evidence is not in their favor, can de-campaign it or misrepresent results (2)	Inclined to serve political interests
		May remain unconcerned about available evidence and play a passive role (1)	

	Establishing structures that can improve uptake of research (*e.g.* knowledge brokers), developing a communication strategy and a community research advisory network (5)		
	Providing stewardship (5)		
	Participating in research (1)		
Parliamentarians/politicians	Demanding implementation of evidence (8)	Focus may differ; if they see that the available evidence does not favor their objectives and may lead to the loss of votes, they will not support the evidence. If they stand to benefit, they will support the evidence (4)	Difference in objectives; technical objectives may differ from political objectives
	Advocating for funding to implement recommendations (4)		
	Mobilizing and disseminating evidence to their communities (4)		
Researchers	Undertaking research (14)	Corruption affects the research community to the extent that they may even provide misleading results (1)	Balancing satisfying academic interest and community needs
	Research users (1)		
Donors	Providing funding for research (16)	May carry out research that does not focus on local needs (3)	Availability of institutionalized platforms for setting research agendas and engaging in KT under strong MoH leadership
	Providing funding for implementation (9)		
		At times they work toward fulfilling agency agendas (2)	
	Undertaking research activities (5)		
		May refuse to fund implementation of certain recommendations, for whatever reasons (1)	
	Encouraging the development of evidence-based polices (2)		
	WHO provides global evidence that guides policy development in countries (2)		
		Can make financial commitments to support implementation of evidence but fail to meet them (1)	
	Implementing research recommendations (1)		
	Influencing the research agenda (1)		
		Failure to contextualize global knowledge (1)	
	May bring in global knowledge (1)		
Private health providers	Using evidence to make investment decisions (1)		Weak internal organization

### CSOs

Almost half of the respondents (n = 10) mentioned the use of research results as a role that CSOs can play in KT. A MoH official remarked that “*civil society, most especially those concerned with health issues, have the capacity to implement research recommendations.*” A donor official stated that “*civil society can mobilize dedicated funding and can actually help to implement research findings*.”

Eight respondents also included advocating with policy makers to implement evidence, developing policies that incorporate evidence, and community mobilization among the roles of CSOs in KT.

A civil society respondent remarked that “*civil society is very key; they can disseminate research, advocate with policy makers, mobilize communities, and liaise with the media*.”

A MoH respondent also emphasized this role, stating that “*civil society, most especially those concerned with health issues, should be involved in most decisions because they have the capacity to mobilize communities to support policy development and implementation*.”

Regarding CSOs undertaking research, one respondent stated that CSOs are limited by their weak position; the MoH does not believe that they have the capacity to undertake high-quality research. A CSO respondent stated, “*Sometimes we have failed to commission studies because if these studies are done by us, the attitude in the MoH is such that they may not believe in them, but if they are performed by WHO or UNICEF, then the big name carries the day. So, many times, we have been discouraged at the pre-research stage*.”

Negative roles that CSOs may play in KT were also mentioned. These roles included undermining evidence that they are not convinced about and causing confusion in the community if they are not given proper information.

One respondent, who is a researcher, articulated challenges facing CSOs in KT, with the exception of religion-affiliated bureaus. This respondent was concerned about their lack of capacity, weak internal organization, and lack of independence. Most CSOs are funded by donors, and some receive funding from government and that compromises their independence. The respondent said, “*Civil society is not a big voice; they are currently just being imposed on us by donors. They lack funding independent of the government and lack technical capacity. They are not organized well enough to engage in policy development, can’t harness their power to influence policy processes, and may not even be educated enough*.”

### Communities

Six respondents expressed the opinion that the communities’ main roles were to demand implementation of evidence and to contribute to the development of the research agenda.

A donor respondent stated that “*communities can advocate for their needs, speaking up when the policy that should be in place is not being implemented.* [They] *can help researchers to better define their questions, and can also demand to know what research is being done and whether it will benefit them*.”

A civil society respondent said that “*communities are the beneficiary of the research; they can demand that evidence is implemented through political leaders like members of parliament, through demonstrations, but they can also contribute to development of the research agenda*.”

One respondent mentioned that this role requires that researchers and policy makers understand how to engage communities. In addition, there must be organized community structures that enable communities to participate in research processes.

However, one researcher raised concerns regarding whether the community was able to play any role in KT, stating that “*the community is dislocated from the main machinery, both in terms of undertaking research but also in policy development. Their contribution is very small, they are not key stakeholders. Power and decision making is very centralized, and even decentralization that was meant to enhance community participation has yet to be achieved. Communities have no political voice, partly because of poverty and lack of education*.”

### The media

A majority of respondents expressed the idea that the role of the media is to disseminate evidence, and also to bring the views of the community to policymakers.

A MoH respondent said that “*the media definitely play a key role in dissemination, informing people about new research findings. They put forward the views of the community*.”

A private for-profit respondent highlighted the dissemination role of the media as “*the media have a positive influence; they are the means of transfer of information, they are a communication channel to the public*.”

A journalist remarked that “*the media act as a public watchdog; they can make something a national issue. They are also a pressure group; they can mobilize for action and also sensitize the public about the research findings. In about ten minutes, knowledge can be transferred instantaneously and simultaneously to the whole world*.”

Respondents identified several negative roles of the media, such as misrepresenting data when evidence is passed on to the media in a poorly packaged form.

A private for-profit respondent said that “*the way you engage in dialogue with the media and the way information is packaged will determine the impact of the media. If information is poorly packaged and poorly passed on, they will misrepresent the evidence. If you don’t want them to provide wrong information, give the correct information*.”

A donor stated that “*the media can have a negative influence if they misrepresent your results or the ideas of your research, e.g. telling the public that they have come to us as guinea pigs in reference to a new research project like a drug trial*.”

A civil society respondent indicated that “*the media can play a negative role by misrepresenting information for their own interests, so you need to bring them on board. We should work hard as professionals to have the media on board; we need to create time for them*.”

In order to minimize misrepresentation, one respondent mentioned the need to carefully select messages and to provide written messages as opposed to verbal communication.

The challenges hampering the effectiveness of the media in KT included not being well organized and communicating with a public that is not strong enough to respond. One researcher stated, “*The media are not very strong. They should be the civil watchdog reporting to the public (…) the public is not organized enough to follow up on what is reported. In this case, it weakens the role of the media. The media should work hand-in-hand with the public, but the public is weak and this also weakens the media. An example is drug thefts that have been reported in papers, but the public has never come up to demand accountability*.”

### Government policymakers

Many respondents (n = 12) stated that the role of government policy makers in KT is to make use of evidence and to identify knowledge gaps. One respondent said that “*government policy makers and technical ministries play a role in policy formulation, but they are also the implementers. They should use evidence to formulate policies*.”

Five respondents mentioned that government policy makers should put in place structures and platforms that can improve the uptake of research and play a stewardship role in the generation and uptake of evidence. A donor respondent stated that “*they should ensure that dialogue takes place, they should take a stewardship role, ensure that research results are discussed and use them for policy development*.”

Some negative roles identified by respondents included ignoring or even misrepresenting evidence that is not in their favor, as illustrated in the following quote by a civil society respondent, “t*hey can play a negative role if the research result is not in their favor. If it is not in their favor they can actually ignore it. The other negative role is they can misinterpret it to suit whatever side they want to lean against*.”

Another negative role indicated by respondents was the inclination to serve political interests irrespective of available evidence. A journalist said that “*they tend to operate in the political domain, which limits them depending on the regime. They are sometimes insensitive to some societal needs, and they become negative on things that should really be supported*.”

### Politicians/parliamentarians

Respondents perceived that the main role of parliamentarians is to ensure implementation by demanding that evidence is implemented (n = 8) and by advocating for funding for its implementation and dissemination (n = 4). A civil society respondent stated that “*parliamentarians can mobilize communities; each parliamentarian comes from a constituency, and can mobilize his/her people to demand the implementation of evidence. But they also play a role in dissemination; they can disseminate evidence to their communities. They also push senior officers within the MoH to implement evidence*.”

Differences in the objectives being pursued underpinned a negative role attributed to politicians. Four respondents mentioned that the priority of politicians is to secure the electorate’s vote; these respondents felt that if the evidence is contrary to this objective, the politicians will not support it.

For example, a MoH respondent said “*People fear to carry out research that is against politicians’ interests, they fear annoying them. If findings are in their favor, they can help you push the implementation of recommendations. However, politicians will stop research implementation if it is against their interests*.”

A private for-profit respondent stated, “*Politicians tend to have inclinations. Policies are twisted towards political interests and in that sense, they can have a negative influence. Political peddling has an impact on how that policy is going to be. If they are open towards evidence and understand the value of research, they can play a positive role*.”

### Researchers

Although the main role of researchers was identified as undertaking research, respondents pointed out that researchers need to perform research in collaboration with beneficiaries like the MoH. Respondents were also concerned about the focus of the research community on satisfying academic interests rather than community needs. In addition, respondents highlighted the issue of corruption affecting the research community, which is understood to occur when funds are provided and inferior-quality research or misleading evidence is produced.

### Donors

Respondents described the role of donors in KT as providing funding for undertaking research (n = 13) and providing funding for implementing research findings (n = 16), as illustrated in the following quotes. First, a donor respondent said,

“*Donors should fund research, although it must be in line with the priorities identified in the research agenda. They should fund dissemination activities. They should also contribute to funding the implementation of recommendations*.”

Second, an MoH respondent stated that “*donors are key for funding, and can provide funds for the implementation of research recommendations*.”

Third, a journalist said, “*When it comes to the generation and use of evidence, donors can comfortably perform in funding*.”

Although undertaking research was identified as a donor role by five respondents, respondents also were concerned about the local relevance of donor-supported research.

A civil society respondent remarked, “*They should not just bring research of their interest. The north-to-south collaboration in research should be fine-tuned to make research more responsive to knowledge gaps as perceived at the local level*,”

A MoH respondent stated that “*donors may impose a research agenda on us that is not our priority*.”

A private for-profit respondent raised the issue of the donor agency agenda overtaking local interests in the research process, stating, “*Donors are sometimes bent towards fulfilling agency agendas. If our priorities are not clear, support may be wasted through duplication and confusion*.”

Almost all respondents (n = 18) stated that stakeholders need to work together in a partnership right from setting the research agenda, through the research or knowledge generation process, dissemination and applying the knowledge. Seventeen respondents indicated that institutionalized platforms bringing together all relevant stakeholders are necessary to ensure that partnerships are effective; they reported that these platforms were lacking, and that the leadership of the MoH in guiding research processes and applying evidence was weak.

A MoH respondent said, “*There is no systematic dialogue between policy makers and researchers. Other stakeholders must be involved as well. The Uganda National Health Research Organization that should coordinate this exercise is still weak*.”

A civil society respondent declared that *“there must be a continuous rapport between researchers and the government. I think when researchers give evidence, that’s where it stops, there must be continuous networking and linkage*.”

Another civil society respondent stated, “*Partnership is very much necessary, the community is not involved, the community puts in little yet all should be included in policy development*.”

These platforms would ensure systematic dialogue. One civil society respondent emphasized that “*we need to have a research advisory network in place. This should bring together researchers, civil society, knowledge brokers, and policy makers. In this way, it would be easy to have a research agenda agreed on by all stakeholders that will be followed and results well disseminated and implemented*.”

## Discussion

Our study has yielded useful insights into the roles of the various stakeholders in KT in Uganda. The literature emphasizes that the roles assigned to stakeholders should correspond to their skills and expertise [38]. The need for systematic and meaningful involvement of stakeholders in partnerships, working in a complimentary manner to achieve a common objective, has also been raised [34]. This goal calls for establishing inclusive platforms with appropriate leadership to bring all stakeholders together. In addition, there must be mechanisms for addressing conflicts of interest and guarding against undue influence. Deslie *et al.* noted that the weaknesses facing various stakeholders must be addressed, their skills must be enhanced, and information must be shared in understandable formats if stakeholders are to play their roles effectively [13].

Respondents noted that the uptake of evidence in public health policy development and implementation can be performed by government policy makers and CSOs. In the case of CSOs, these findings are similar to what has been identified in literature [13,16]. Their ability to mobilize funding and implement health programs can enhance their contribution to implementing evidence. However, other researchers caution that their capacity to engage in policy development is limited, especially in LICs, and questions about the legitimacy of their policy positions and accountability have been raised [39]. Policy makers serve a leadership role in policy development and are definitely central to the uptake of evidence in policy development. However, literature has shown that they often work under severe time constraints and political pressure, which may not allow enough time for the application of evidence [40]. This issue requires the balancing of time pressures, timely provision of evidence, and implementing decisions. The potentially superficial understanding of the subject matter by policy makers, who are often responsible for several areas, is also a documented challenge [40]; researchers can minimize the effect of this superficial understanding by summarizing evidence in brief, digestible formats.

The dissemination of evidence was identified as a role that can be played by the media, CSOs, and parliamentarians. In the case of the media, our respondents highlighted the risk of misrepresenting evidence and a weak public as compromising the media’s effectiveness. The role of the media in disseminating evidence has been documented, and researchers have noted that more contact with the media will help strengthen the media’s capacity for science reporting [41]. Indeed, an earlier study in Uganda noted the poorly coordinated efforts of researchers and communities to reach the media, concluding that the media is a powerful ally that is under-utilized by public health professionals [42]. The literature emphasizes the importance of presenting evidence to CSOs in a clear and simplified format if they are to effectively disseminate evidence; in addition, regular updates need to be provided throughout the research process [16,43]. These actions will safeguard against the negative roles identified in this study, especially misrepresentation of evidence and the creation of confusion in the community. Although the literature indicates that researchers can engage in dissemination, our respondents did not identify this role. Researchers may instead focus on the generation of user-friendly research products and improving the engagement with the media and with CSOs. This focus reflects the results of studies of the process of translating research on male medical circumcision and prevention of mother-to-child transmission into policy; Ssengooba *et al.* reported that researchers were found to be media “shy” [26]. Some studies have reported that researchers can also act as policy entrepreneurs by organizing themselves into networks that can engage policy makers. In instances in which this strategy was successful, some of the policy makers had research backgrounds [33]. This strategy was not mentioned by our respondents.

Advocating for the implementation of evidence, exerting pressure on policy makers, and mobilizing communities were identified as roles for politicians/parliamentarians and CSOs. These roles are similar to those previously identified in the literature [13,15,16]. CSOs and parliamentarians are nearer to communities and are supposed to represent community interests. CSOs have been shown to be effective advocates once empowered with evidence; they have been able to mobilize communities to demand government accountability and to encourage donors to focus on country priorities [13]. Respondents in our study noted the need for CSOs to be funded outside of the government to ensure independence. However, the dependency of CSOs on donors to finance their operations, especially in LICs, will remain a constraint in that CSOs may be hesitant to take a position that is against the position of their funders [17]. Regarding politicians/parliamentarians, effective realization of their roles continues to be challenging given the nature of political systems in LICs. Political ideology and the tendency to respond to the demands of constituencies or electorates, irrespective of the evidence, are among the challenges that must be overcome [27]. Young previously highlighted the need to better understand the political processes of LICs in order to understand the role of evidence in policy making [27]. The community has been noted to be able to exert pressure on policy makers to respond to evidence [44], which can be enhanced through effective dissemination of research to communities.

Although previous research revealed that undertaking research is a role that could be played by CSOs [13,16], it was not identified as a major role of CSOs in our study. This result reflects the often weak linkage between CSOs and the research community and the limited scope of their research, which is mainly limited to evaluation of their programs [13]. The capacity of CSOs to engage in research can only be encouraged if investments can be made with respect to training, mentorship programs, or through formal partnerships between communities and universities that link CSOs with academic researchers. Community participation in setting the research agenda, which was highlighted in this study, has also been identified by other researchers [45,46], who noted that this participation may improve the responsiveness of research to community priorities. Several challenges confronting researchers were identified by our respondents, including the risk of disproportionate focus on academic interests and failing to address community priorities; these challenges were also pointed out by other researchers [43,44]. In addition, the tradeoff between producing high-quality research and the time-constrained nature of the policy-making process is often difficult to achieve.

In our study, funding research and implementing evidence were identified as roles played by donors, a finding similar to other studies [3,21]. The funding role of donors in KT has long been seen as potentially supportive and disruptive at the same time. In some instances, funding has been used to influence research agendas and policy development in LICs [42]. For example, Burris *et al.* reported that the funding requirements of donors in Ghana were the strongest impetus for the uptake of evidence in guideline formulation for HIV care [44]. Similarly, the decision to change Uganda’s malaria treatment policy was heavily influenced by the availability of funding from the Global Fund against AIDS, Tuberculosis, and Malaria [47]. Many LICs depend heavily on foreign aid to fund health services, allowing donors to exert undue influence on research processes and programming decisions. Some researchers have highlighted the need to better understand the interface between development agencies and national processes in LICs [3]. Donor funding can definitely contribute positively to research and policy development in LICs, but governments must be able to effectively fulfill their stewardship role [48]. This undue influence of donors has been controlled to some extent when governments have established structures to develop research agendas through inclusive partnerships [34]. We were surprised to note that no respondent mentioned funding of research and implementation of evidence as a role for governments.

In our study, there was also no mention of professional bodies and informal policy networks as stakeholders in KT. Reasons for this omission may include the relative weakness of professional bodies in Uganda and the fact that they are not much known outside the MoH. In addition, professional bodies are not very active in public-health policy making, which was the focus of this study, as has been reported for several African countries [49]. Their role is more pronounced in the development of clinical practice guidelines, and positive contributions have been documented [33]. Knowledge brokers were not identified as stakeholders by the respondents of the present study, perhaps because this is a relatively new concept; there are currently no knowledge brokers active in Uganda.

In this study, government policy makers, CSOs, the media, parliamentarians/politicians, communities, researchers, and donors were identified as stakeholders in KT for public health policy in Uganda. The roles played by these stakeholders and the challenges that remain to be overcome are summarized below.

### Role: Uptake of evidence in public health policy development and implementation

For CSOs to effectively engage in policy development, their skills must encompass navigating the political terrain, engaging policy makers, and networking, which call for high levels of internal organization and independence [16,39]. Uptake of evidence by policy makers could be improved by recruiting advisors and/or establishing think tanks to synthesize evidence in simple, digestible formats, as already attempted in some LICs [27]. The links among policy makers, researchers, and CSOs have long been known to be potentially beneficial, and should be strengthened [5,24] to enable systematic information-sharing and synergy in evidence implementation [39]. Policy makers need to put in place the institutional frameworks and platforms for engagement that will enhance greater ownership and application of evidence by policy makers and politicians [11].

### Role: Dissemination

The risk of misrepresentation of evidence by the media must be minimized through improved dissemination of evidence, preferably in written formats rather than verbal communication alone. The literature underscores the critical impact of communicating evidence in a clear, conclusive, and accessible way [16,43]. Further, platforms need to be established for systematic engagement and information-sharing among researchers, policy makers, and the media. In order to improve the impact of the media, there must be parallel efforts to strengthen the public’s response to media messages.

For CSOs to strengthen their contributions as disseminators of evidence, evidence must be presented to CSOs in clear and adapted formats, and regular updates should be provided. Similarly, parliamentarians need to be armed with accurate information through targeted dissemination and regular updates.

### Role: Advocating for the implementation of evidence, exerting pressure on policy makers, and mobilizing communities

To achieve this role, CSOs need to be independent and funded independently of government channels. The links between parliamentarians and community structures on the one hand and between parliamentarians and researchers on the other must be strengthened to ensure that politicians are provided with accurate information. Further, there is a need for mechanisms of targeted dissemination of evidence to communities in simplified and appropriate formats. Platforms for researchers, policy makers, and communities to effectively engage with communities and for organizing communities to be able to engage in policy processes must be put in place [45].

### Role: Undertaking research

Structures must be established to enable communities to participate in research processes; these structures should include links between communities and researchers. Communities must be organized, and consultative mechanisms need to be established for them to engage in setting research priorities. For researchers, institutional platforms should be established between researchers and policy makers so that researchers appreciate both the policy-making process and the pressure under which policy makers work. At the same time, these platforms could help policy makers to better appreciate the research process and the constraints faced by researchers. Such platforms provide learning opportunities for all actors [26]. The failure to focus on community needs and local priorities can be addressed through mechanisms for inclusion in the setting of research priorities.

### Role: Funding research and implementation of evidence

In order to focus donor support on the priorities of the country, governments need to establish structures to develop research agendas through inclusive and participatory partnerships. The advocacy and community-mobilization roles of CSOs could also be employed to ensure that donor funding supports locally identified priorities.

### Study limitations

In the interviews, no references were made to either a specific research study or to actual policy, which may have influenced the respondents’ reflections on the KT process in Uganda by keeping the discussion at a more abstract level. However, it could also be argued that mentioning a specific policy could have induced the respondents to be more anecdotal in their responses. We also note that some respondents may have dual roles in KT. For instance, a researcher may be working for civil society, and therefore represent two stakeholder groups at the same time. The influence of these dual roles was not explored in this study; it could be considered a strength as much as a weakness of this investigation, in that this influence may have enriched the reflection more than it limited it. We did not use the Delphi method to identify the shared views of the various stakeholders and to reach theoretical saturation. While such a process may have its benefits, we believe that in-depth interviews allowed more efficient exploration of the views of the various groups, which in a second phase can still be presented to a wider group through a Delphi process. Finally, this study focused on KT in reference to public health policies and may not be representative of other fields such as clinical interventions.

## Conclusions

Previously, Sauerborn *et al.* emphasized the importance of considering the needs of various stakeholders [10]. We argue that there is a need to take into consideration the roles that the various stakeholders are best positioned to play in KT. Linkages must be built and necessary platforms put in place to achieve synergy among stakeholders in KT. At the same time, the challenges faced by the various stakeholders need to be overcome, and relevant capacities must be built if they are to play their roles effectively.

## Endnotes

^a^ In the health sector in Uganda, policy development and implementation of health programs have been undertaken within a partnership under the sector-wide approach since 2000 [35]. The process of policy development usually begins with discussions within technical working groups comprised of government officials, donors, civil society representatives, and private for-profit health providers. Technical working groups propose options that are discussed further in the HPAC, the policy advisory body of the health sector. The HPAC consists of senior government officials from the central and district levels and representatives from donor agencies and the private not-for-profit and private for-profit sectors. The HPAC makes final decisions regarding the adoption of policy options.

## Abbreviations

CSO: Civil society organization; HPAC: Health policy advisory committee; KT: Knowledge translation; LICs: Low-income countries; MoH: Ministry of health; MRT: Middle range theory; WHO: World health organization

## Competing interests

The authors declare that they have no competing interests.

## Authors’ contributions

JNO contributed to the conception and design of the study, to data collection, analysis, and interpretation, and led the drafting of the manuscript. BM contributed to data interpretation and to drafting of the manuscript. DKM participated in data analysis, interpretation, and drafting of the manuscript. FS participated in study conception and design, data analysis and interpretation, and drafting of the manuscript. JM participated in study conception and design, data analysis and interpretation, and drafting of the manuscript. VCS participated in interpretation of the results and drafting of the manuscript. BC contributed to study conception and design, data analysis and interpretation, and drafting of the manuscript. All authors read and approved the final manuscript.

## Pre-publication history

The pre-publication history for this paper can be accessed here:

http://www.biomedcentral.com/1472-6963/13/324/prepub
